# Supplementary material for: Chromosome Number and Genome Size Evolution in Brasolia and Sobralia (Sobralieae, Orchidaceae)
Source: Int J Mol Sci. 2022 Apr 1;23(7):3948. doi: 10.3390/ijms23073948 (PMC8999598; doi:10.3390/ijms23073948)
Supplement: Supplementary file 1 [file ijms-23-03948-s001.zip › ijms-1639239-supplementary.pdf]

Baranow, P.; Rojek, J.; Dudek, M.; Szlachetko, D.; Bohdanowicz, J.; Kapusta, M.; Jedrzejczyk, I.; Rewers, M.; Moraes, A.P. Chromosome Number and Genome Size Evolution in *Brasolia* and *Sobralia* (Sobralieae, Orchidaceae). *Int. J. Mol. Sci.* 2022, *23*, x. https://doi.org/10.3390/xxxxx

Supplementary Materials: The following supporting information can be downloaded at: www.mdpi.com/xxx/s1,

Figure S1**:** Maximum Likelihood (A) and Bayesian Inference (B) phylogenetic trees based on *mat*K-*trn*K and ITS markers.

Figure S2. Chromosome complement of *Brasolia* and *Elleanthus* species. (a) *B. altissima* (2*n* = 46), (b) *B. cattleya* (2*n* = 42), (c) *B. dichotoma* (2*n* = 46), (d) *B. flava* (2*n* = 46), (e) *B. hirtzii* (2*n* = 44), (f) *B. portialle* (2*n* = 48), (g) *E. brasiliensis* (2*n* = 50), (h) *E. crinipes* (2*n* = 50). Each chromosome is indicated by a number. Bar in h correspond to 5 µm.

Figure S3. Chromosome complement of *Sobralia* species. (a) *S. bimaculata* (2*n* = 44), (b) *S. decora* (2*n* = 42), (c) *S. decora* (2*n* = 44), (d) *S. fimbriata* (2*n* = 46), (e) *S. macrantha* (2*n* = 48), (f) *S. powelli* (2*n* = 44), (g) *S. vallecaucana* (2*n* = 48), (h) *S. wilsoniana* (2*n* = 48), (i) *S. yauaperyensis* (2*n* = 46), (j) *S. crocea* (2*n* = 44), (k) *S. fragans* (2*n* = 42), (l) *S. gloriosa* (2*n* = 48), (m) *S. luerorum* (2*n* = 48), (n) *S. rosea* (2*n* = 48). Each chromosome is indicated by a number. Bar in h correspond to 5 µm.

Table S1. GenBank accession numbers of Internal Transcribed Sequences (ITS) and *mat*K sequences used for the complete phylogenetic reconstruction. A total of 57 individuals from 53 species distributed in four genera were used: 11 *Brasolia*, four *Elleanthus*, 34 *Sobralia*, and four *Neottia* species, used as outgroup.

Table S2. Expectations probabilities considering the model Constant Rate without duplication for both phylogenetic analysis. The main mechanism of chromosome number rearrangment is indicated in bold (higher expectation on number of events).

Table S1. GenBank accession numbers of Internal Transcribed Sequences (ITS) and *mat*K sequences used for the complete phylogenetic reconstruction. A total of 57 individuals from 53 species distributed in four genera were used: 11 *Brasolia*, four *Elleanthus*, 34 *Sobralia*, and four *Neottia* species, used as outgroup.

**Taxa**	**ITS**	***mat*K**
Sobralia andreae	KT923834.1	KT428828.1
Sobralia atropubescens	KT923842.1	KT428835.1
Sobralia bimaculata	KF612992.1	KF700346.1
Sobralia callosa	KT923833.1	KT428827.1
Sobralia cobanensis	KF612993.1	KF700347.1
Sobralia crocea	HM854644.1	HQ329050.1
Sobralia decora	KF612994.1	KF700348.1

Sobralia fragrans	KF612997.1	KF700351.1
Sobralia fimbriata	HM854631.1	HQ329037.1
Sobralia gloriosa	HM854629.1	HQ329035.1
Sobralia klotzscheana	HM854643.1	HQ329049.1
Sobralia lancea	KT923844.1	KT428837.1
Sobralia lindleyana	HM854624.1	HQ329030.1
Sobralia luerorum	MW628176.1	MZ170069.1
Sobralia macrantha	HM854651.1	HQ329058.1
Sobralia macrophylla	KT923840.1	KT428833.1
Sobralia maduroi	KT923828.1	KT428822.1
Sobralia cf. madisonii	HM854634.1	HQ329040.1
Sobralia melantothrix	KT923842.1	KT428835.1
Sobralia mucronata	KF612996.1	KF700350.1
Sobralia powelli	KF612986.1	KF700340.1
Sobralia pulcherima	KF612992.1	KF700346.1
Sobralia rogersiana	KT923830.1	KT428824.1
Sobralia rosea	KT923827.1	KT428821.1
Sobralia sessilis	MW628177.1	MZ170070.1
Sobralia triandra	KT923841.1	KT428834.1
Sobralia tricolor	KT923843.1	KT428836.1
Sobralia undatocarinata	KT923829.1	KT428823.1
Sobralia vallecaucana	KF613001.1	KF700355.1
Sobralia virginalis	KT923836.1	KT428829.1
Sobralia warszewiczii	KT923838.1	KT428831.1
Sobralia wilsoniana	KT923837.1	KT428830.1
Sobralia withneri	KT923839.1	KT428832.1
Sobralia yauaperyensis	HM854621.1	HQ329027.1
Brasolia altissima	HM854648.1	HQ329054.1
Brasolia caloglossa	KT923826.1	KT428820.1
Brasolia cattleya	KT923818.1	KT428817.1
Brasolia dichotoma	KT923814.1	KT428818.1

Brasolia dichotoma	HM854647.1	HQ329053.1
Brasolia flava	KT923817.1	KT428816.1
Brasolia flava	KT923816.1	KT428815.1
Brasolia flava	KT923815.1	KT428814.1
Brasolia flava	HM854637.1	HQ329043.1
Brasolia hirtzii	HM854641.1	HQ329047.1
Brasolia hirtzii	HM854640.1	HQ329046.1
Brasolia portillae	KT923813.1	KT428812.1
Brasolia portillae	KT923812.1	KT428811.1
Brasolia roezlii	KT923824.1	KT428819.1
Elleanthus brasiliensis	MZ153093.1	MZ170071.1
Elleanthus crinipes	MZ153094.1	--------
Elleanthus oliganthus	MH656420.1	MH733930.1
Elleanthus purpureus	HM854582.1	MH733936.1
Neottia acuminata	MH808060.1	KF695166.1
Neottia cordata	KX167869.1	KX677756.1
Neottia nidusavis	KX167909.1	AY368419.1
Neottia pinetorum	KT338759.1	KF262100.1

Table S2. Expectations probabilities considering the model Constant Rate without duplication for both phylogenetic analysis. The main mechanism of chromosome number rearrangment is indicated in bold (higher expectation on number of events).

**Total number of:**	**Maximum Likelihood**	**Bayesian Inference**
gain events (ascending dysploidy)	30.9113	15.2518
**loss events (descending dysploidy)**	**49.1808**	**37.0653**
duplication events	0	0
demi-duplications events	0	0
