# Supplementary figures and images for: Chromosome Number and Genome Size Evolution in Brasolia and Sobralia (Sobralieae, Orchidaceae)
Source: Int J Mol Sci. 2022 Apr 1;23(7):3948. doi: 10.3390/ijms23073948 (PMC8999598; doi:10.3390/ijms23073948)

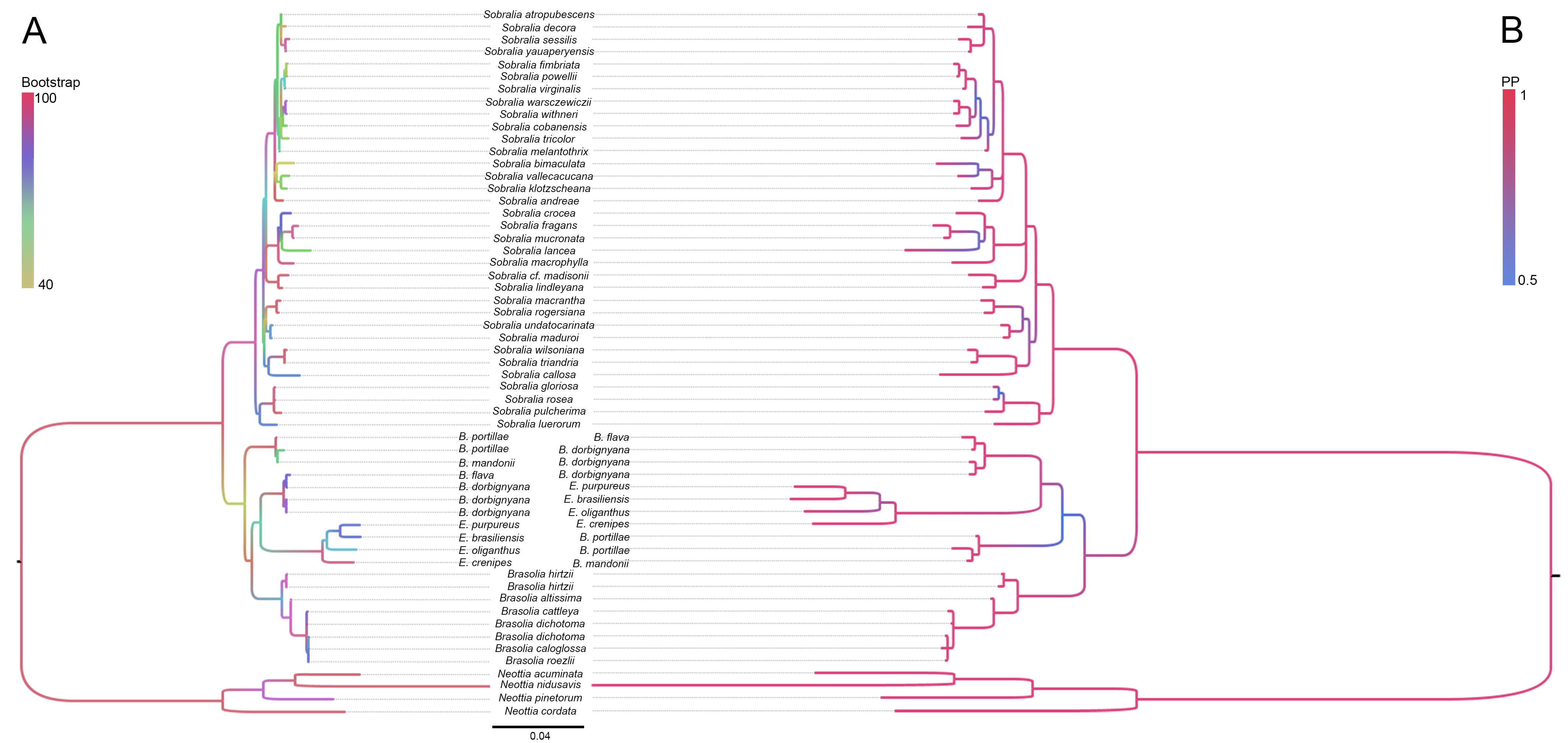

Supplement: Supplementary file 1 [file ijms-23-03948-s001.zip › Figure S1.tif]

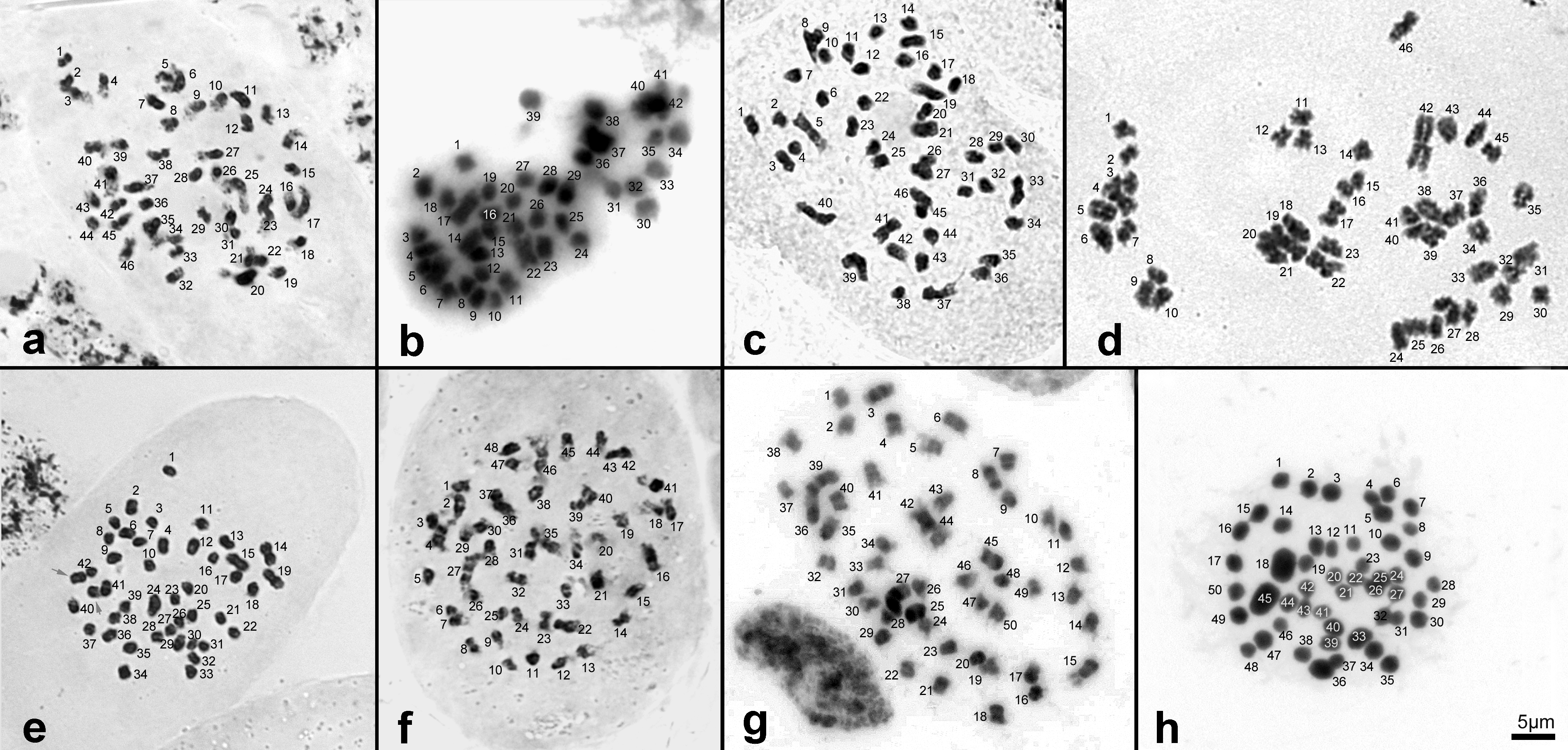

Supplement: Supplementary file 1 [file ijms-23-03948-s001.zip › Figure S2.tif]

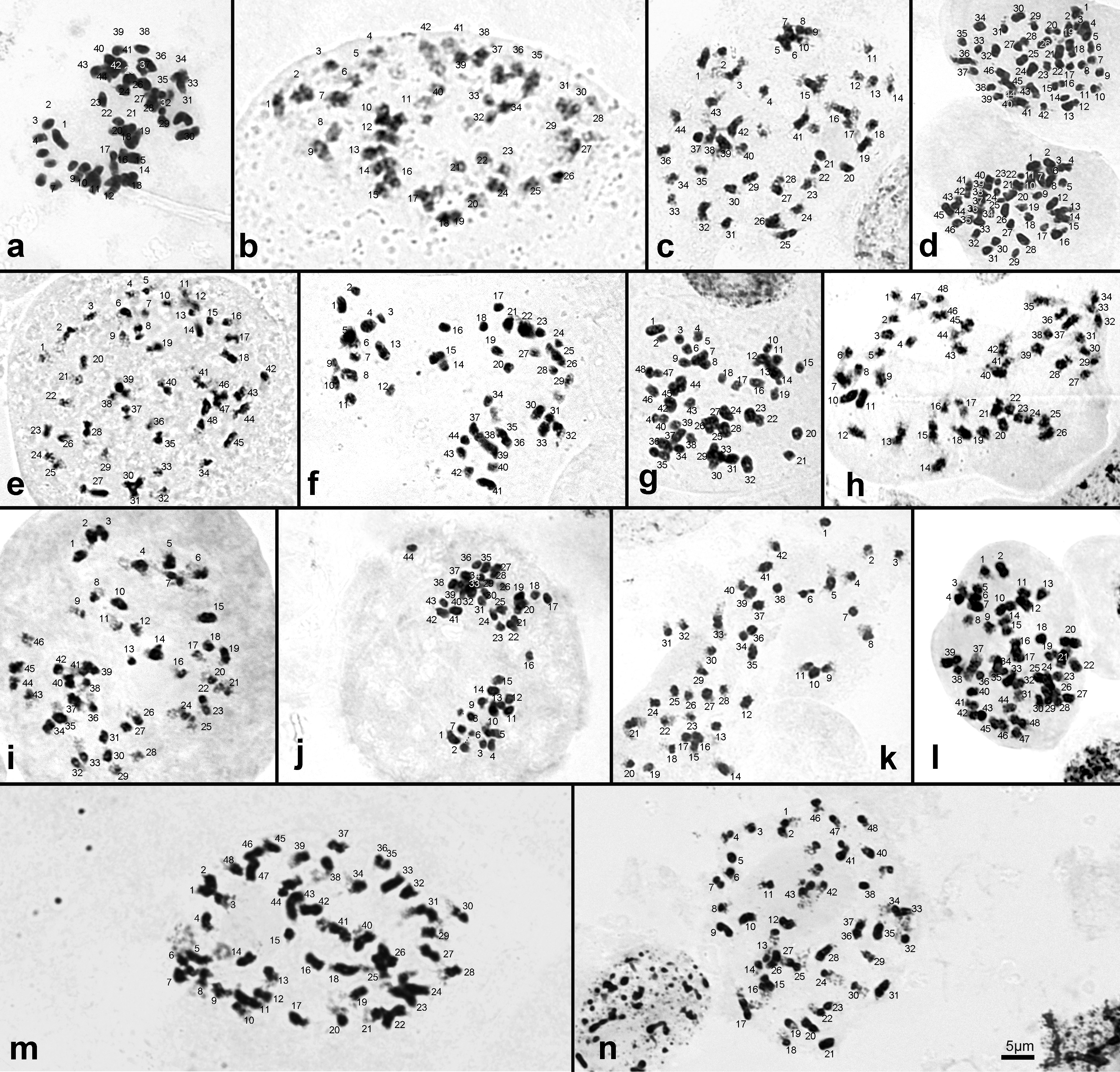

Supplement: Supplementary file 1 [file ijms-23-03948-s001.zip › Figure S3.tif]
